# Medical Opinion and Movement

**Published:** 1909-08-28

**Authors:** 


### Medical Opinion and Movement.

Dr. E. K. Macomber has advocated for a decade the use of Dilating Bags for the Prevention of Perineal Lacerations during labour. His method is given as follows in the *American Journal of Obstetrics*. After the cervix has dilated to the size of a silver dollar or a little larger, and labour pains are active, the dilating bag of Champetier de Ribes, or one of its modifications, is boiled, lubricated, and introduced into the vagina; it is then slowly filled with a warm antiseptic solution and the bag left to be expelled, being held during each pain to prevent rapid expulsion, which might cause lacerations. It is claimed that by this means the vagina is gradually dilated in such a way that the second stage of labour is materially shortened: in many cases the fœtal head emerges from the vulva immediately after the bag is expelled. At the same time the presence of the bag stimulates the secreting glands of the vagina, and thus keeps the passage well lubricated. Furthermore, the pains do not so frequently become inefficient during the second stage; and if they do, slight traction on the bag will immediately bring on a pain. By this method it is said that instrumental deliveries are very largely avoided, as well as the lacerations so common in spontaneous delivery. The direction of the dilating force is also towards the outlet of the vulva instead of towards the centre of the perineum. Several American obstetricians who have tried this plan report favourably on it, though one or two evidently have doubts about recommending it unless the manipulator is thoroughly *au fait* with antiseptic routine.

The Induction of Labour at Term as a matter of routine is advocated by Dr. A. H. Wright in a paper which has been read before the Toronto Academy of Medicine. The author commences by reporting a case in which pregnancy went to five weeks beyond term, and resulted in the birth of a child of 12½ lbs. weight, which lived only half an hour, and resulted in four months' confinement to bed for the mother; and he believes that such cases of unduly prolonged gestation are not uncommon. Taking two hundred and seventy days as the normal term of pregnancy, he formulates recommendations to induce labour in all cases within two or three days after the expected date of confinement, whether there are any symptoms of approaching labour or not. To plug the vagina by the Schauta method, with especial care to pack the vault tightly. To allow the patient to get up and go about, after plugging, if she chooses. In twenty-four hours to remove the tampon and put in a fresh one; and if this does not induce labour within a further twenty-four hours, to pass a bougie into the uterus and wedge it up with a third tampon. As a matter of experience, the bougie is unnecessary in a large proportion of cases, and when this is the case it is evident that from the point of view of aseptic midwifery there is no objection at all to the procedure.

A very interesting study of the question of the usual mode of infection in pulmonary tuberculosis is contributed to *La Semaine Médicale* by Dr. J. Lehrmitte. For some time the view has been generally accepted, as first propounded by Koch, Baumgarten, and others, that in ordinary pulmonary tuberculosis infection takes place through the air passages. Pathological anatomy confirmed this view, and showed that in the early stages the morbid lesions were purely alveolar and resulted from the irritation and necrosing action of the tubercle bacilli at the seat of inoculation. The further extension of the disease depended upon the conditions of resistance of the organism. More recently, however, this inhalation theory has been seriously contested, and it has been sought to prove that the intestinal and lymphatic channels were the more usual routes of infection to the lungs. This hypothesis has been based upon the experimental researches of Chauveau, Von Behring, Calmette, and Guérin, by which it has been shown that the ingestion of tubercle bacilli in animals may lead to tubercular lesions of the lungs. From these facts Von Behring has developed the view that a pulmonary tuberculosis in adult life may owe its origin to an intestinal infection that has been contracted during childhood and that has remained latent for some time before setting up the pulmonary lesion.

These theories Dr. Lehrmitte proceeds to contest from two points of view. In the first place the author points out that the pulmonary lesions obtained in animals as a result of experimental injection of the bacilli are of a totally different type from the ordinary chronic pulmonary tuberculosis in man. They correspond to the scattered miliary type of disease, such as is found in conditions of general tuberculosis, in which the bacilli have gained access to the blood-stream. Such experimental results, therefore, in no way justify the supposition that chronic pulmonary tuberculosis can arise in this way. Dr. Lehrmitte then discusses the possible channels by which the bacilli may find their way to the pulmonary tissues. Theoretically they might be carried there either by the lymphatics or by the blood-stream, but actually the only obvious course from the intestines appears to be a mixed one—namely, the mesenteric lymphatics to the thoracic duct, and so into the blood-stream by the subclavian vein, the superior vena-cava, the right side of the heart, and the pulmonary artery. But such an infection *via* the blood-stream does not lead to chronic localised lesions in the lungs or elsewhere, but always to conditions of general tuberculosis, with scattered tubercles, such as have been found in the experiments already referred to. Moreover, pathologically intestinal or mesenteric lesions are of the rarest occurrence in ordinary pulmonary tuberculosis. Dr. Comby, reporting on 525 autopsies of children affected with tuberculosis, has not found a single case in which the primary lesion was in the intestines or mesenteric glands.

It must be admitted, however, that concomitant affections of the glands around the upper part of the alimentary tract, the tonsils, adenoid tissue, and cervical glands do occur with pulmonary tuberculosis. From these parts also the only probable route to the lungs appears to be *via* the lymphatics into the jugular and subclavian veins, and so by the blood-stream to the lesser circulation, and the objections to a pulmonary infection of the ordinary type by this channel have already been stated. It has therefore been supposed that infection may take place entirely through the lymphatic channels, and for this purpose anastomoses have been hypothesised between the cervical, subclavian, and pleural lymphatics, or between the cervical and mediastinal lymphatics. The most careful anatomical researches of Dr. A. Most by means of injections under pressure of coloured liquids into the lymphatics have demonstrated that no such anastomoses exist, that there is no connection between the cervical, subclavian, pleural, and mediastinal lymphatics, by which bacilli or other infective material could pass from the one to the other. Similarly he has shown that there is no connection between the mesenteric, hepatic, or diaphragmatic lymphatics on the one hand, and the pleural or mediastinal lymphatics on the other. The whole theory, therefore, appears to be based upon false suppositions, and the inhalation theory of infection for ordinary chronic pulmonary tuberculosis appears to be the only one feasible.

The coincidence of pulmonary tuberculosis with cervical adenopathies may be explained in the same way as coincidence with other tubercular lesions—for instance, in the joints. Finally, Dr. Lehrmitte discusses the causes which underlie the predilection of the disease for the apex of the lung. Most authors explain this predilection on the ground of deficient expansion of the upper part of the thoracic cavity, causing stagnation and deficient aëration. The expansion of the lungs in deep inspiration causes an increased flow of blood in the vessels, and this would be less marked at the apex, corresponding to the deficiency of expansion. In expiration, too, the contrary takes place, and, in addition to the expulsion of air by the elastic retraction of the lung, there is an expression of the lymphatic vessels. These latter form the filter into which pass the germs and dust from the respiratory epithelium. At the apex, therefore, where this expression of the vessels would be less active, the bacilli have better opportunity to accumulate, settle, and set up a tubercular lesion.

MM. A. Calmette and C. Guérin claim to have found two practical means by which they are able to differentiate Bovine Tubercle Bacilli from the human type. In the first place the bovine bacilli have the characteristic of growing well in the presence of glycerinated ox bile, whereas the human type cannot be cultivated in this way. And again the goat is sensitive to the bovine type, and intramammary injection of the bovine bacilli leads to a definite local development of the disease as well as tuberculous deposits in other parts of the body, while the human bacilli are more or less innocuous to the goat. In a communication on the subject to the Académie des Sciences these authors cite numerous cases in which the origin of the tubercle bacilli was confirmed by these tests, and they have proved the value of these tests by a number of experiments in which the nature of the bacilli was known. In cases in which the bacilli were from the sputum of the tuberculous patients, intramammary injection in goats either had no effect or only produced a little induration of the retromammary glands or small calcified nodules.

Dr. Dahlgren, of Upsala, draws attention to a clinical condition which has all the appearance of a Peritonitis due to perforation of a gastric ulcer, but on opening the abdomen no such lesion is found, and the peritonitis, generally localised, is caused by the propagation of infection through the wall of the stomach. He gives details of two cases of the kind which have come under his notice. The first case was that of a girl aged 20, who had suffered for some time from symptoms of gastric ulcer, and suddenly developed all the symptoms of a peritonitis, with fever and a rapid pulse. On exploration the peritoneal cavity contained serous fluid and considerable injection of the blood-vessels. There was some infiltration and thickening of the gastric wall near the pylorus, but no perforation. Gastrojejunostomy was performed, and the patient made a good recovery. The second case was that of a man aged 33, who also developed acute abdominal symptoms pointing to a local suppurative peritonitis due to perforated gastric ulcer. Operation showed a localised abscess behind the lesser omentum, with adhesions between the liver and stomach, but there was only a congested condition of the vessels of the stomach wall and no ulcer. In both cases then the primary lesion appears to have been in the gastric mucous membrane, an infective process spreading through the wall of the stomach to the peritoneum, probably *via* the lymphatic channels.

Dr. A. von Lichtenberg has made some careful observations on the condition of the lungs and heart after Abdominal Section, and according to his report some slight affection of the lung, bronchitis or pneumonic congestion, is much more common than is usually supposed. These observations were made upon 100 patients of both sexes and all ages up to 70. The nature of the abdominal operations was varied, including those for hernia, appendicitis, gastric and biliary affections, etc., and likewise the anæsthetic—ether, chloroform, local and spinal anæsthesia all being used. The author examined the patients both before and after operation, and found that of the 100 patients after the operation 35 showed physical signs corresponding to a pneumonia, 38 developed bronchitic signs, and only 27 remained free from any change in the respiratory organs. It would appear from these observations that post-operative lung complications of a transient nature are much more common than is usually supposed, and that they are completely overlooked in a large proportion of cases. Dr. Von Lichtenberg suggests that slight elevations of temperature during the first few days after operation are frequently due to such pulmonary affections. There was no relation between these lung affections and the kind of anæsthetic, and the author thinks that the pneumonic signs observed were embolic in origin. Observations on the blood-pressure showed that after a general anæsthetic there was a fall in the blood-pressure which lasted generally twelve or fourteen days or even longer, but after local anæsthesia there was a tendency for the blood-pressure to rise.

An interesting contribution is made by Dr. V. Courtellemont, of Amiens, to *La Semaine Médicale* on what he terms the Quantitative Impermeability of the Kidneys as distinguished from the recognised qualitative impermeability for sodium chloride, urea, or toxins in conditions of Bright's disease. By quantitative impermeability he means the disability of the kidneys to excrete water, and he maintains that this is a characteristic of certain renal affections and is distinguished clinically by dyspnœa often assuming the nature of intense asthmatic attacks. There is, in fact, in these patients a coefficient of capacity for liquids which can be tolerated by the organism but must not be exceeded. This coefficient of capacity or of ingestion is a little more than the amount of urine excreted and is determined by the relation of the amount of liquid ingested to the urine excreted. The difference between these quantities should not exceed 200 or 300 grammes, which it may be supposed are got rid of by other channels. If this coefficient is exceeded, fluid accumulates in the organism and dyspnœa supervenes. The coefficient varies for different cases and should always be determined by reducing the liquid ingested to within about 200 grammes of the urine excreted. In certain cases it may be necessary to reduce the fluid to as little as 800 or 700 grammes. The restriction of fluid in cases of anasarca and cardiac distress is already in common practice, but apart from such conditions the administration of fluids has been considered to assist the elimination of toxic bodies in the blood. If, however, the kidneys cannot cope with the fluid presented, as the author points out, only harm ensues. But the determination of this coefficient of renal capacity for fluid lays down a rational principle for guidance in these cases. The author is of opinion that the dyspnœa is occasioned by an excitation of the pulmonary capillaries by abnormal repletion of the lesser circulation, but he also suggests that it may be an auxiliary means of getting rid of the excess of fluid by increasing the elimination of water vapour through the lungs.

A few years ago Roch pointed out that, in cases of Aortic Insufficiency, capillary pulsation might manifest itself in the eye by variations in size of the pupils occurring synchronously with the heart-beat. Recently Landolfi, of Naples, has studied this question, and finds that the sign is present only when the heart-lesion is one of pure aortic regurgitation, and when marked hypertrophy of the left ventricle and a pronounced Corrigan's pulse are concomitants. The patient should be examined in the upright position, and in order to render the sign more obvious it is usually necessary to employ such artifices as making the patient run, compressing the femoral arteries or the abdominal aorta, and previously administering digitalis. These alternate contractions and dilatations of the pupil can be explained by considering the condition of the iris during systole and diastole respectively. The energetic systolic propulsion of the blood causes hyperæmia of the iris with consequent myosis. During diastole the rapid return of blood towards the heart leads to anæmia of the iris with mydriasis. The phenomenon is therefore a confirmation of the researches of Mosso, who maintained that there exists a relation between the state of the peripheral vessels and that of the pupil.

A somewhat rare complication of Typhoid Fever was reported by Widal at a recent meeting of la Société Médicale des Hôpitaux. The patient, a young man aged 20, was attacked by sudden and complete blindness on the morning of the ninth day of the disease. No history of previous ocular disturbance could be obtained. Ophthalmoscopic examination of the fundi revealed the presence of bilateral œdema of the papilla, with marked venous dilatation. Lumbar puncture was performed immediately, and an exit given to clear fluid, which appeared to be under extreme tension. No albumin or cellular elements could be discovered in the cerebro-spinal fluid. The operation was followed in two hours by marked amelioration of the ocular condition, the improvement continuing until in a few days a complete cure resulted. The fundi were then found to have returned to their normal appearance.

We recently drew attention in an annotation to the Therapeutic Value of Oysters in cases of Tuberculosis, as well as to their use on the Continent as convenient vehicles for the administration of sea-water medicinally. Brintet, of Lyons, has just published a study of the conditions which may follow the ingestion of contaminated oysters, conditions which vary from slight gastro-enteritis to typhoid fever. He finds that as a rule the percentage of consumers of contaminated shell fish attacked is high. The incubation period is a long one, sometimes extending to as much as thirty hours after ingestion. The symptoms of intoxication vary greatly in different cases, but vomiting, colic, and diarrhœa are constantly present. When typhoid fever occurs it is of long duration, and pyrexia is usually high. In one case, that of a child of eight, a high temperature lasted for over forty days. Bacteriological examination rarely reveals the presence of the bacillus of Eberth, but in all the oysters examined the B. coli communis and a large number of the bacilli of putrescence were found. The author cites one interesting case in which a breast-fed child was attacked by severe gastro-intestinal disturbance several hours before the mother herself experienced any discomfort, a fact which tends to show that the period of incubation is shorter in more susceptible persons, as well as that the toxin is excreted in the milk.

